# Transition and Transfer in Adolescence and Emerging Adulthood in Type 1 Diabetes: A Qualitative Study of Parents' Experiences

**DOI:** 10.1002/nop2.70634

**Published:** 2026-06-11

**Authors:** Sara Olsson, Åsa Carlsund, Madeleine Blusi, Julia Otten, Elena Lundberg, Ingela Lavin, Åsa Hörnsten

**Affiliations:** ^1^ Department of Nursing Umeå University Umeå Sweden; ^2^ Department of Public Health and Clinical Medicine Umeå University Umeå Sweden; ^3^ Department of Paediatrics, Institution of Clinical Science Umeå University Umeå Sweden; ^4^ Department of Paediatrics Umeå University Children Hospital Umeå Sweden

**Keywords:** adolescence, adult care, emerging adulthood, experiences, nursing, paediatric care, parents, transfer, transition, type 1 diabetes

## Abstract

**Aim:**

To describe experiences of being a parent of a child with type 1 diabetes during adolescence and emerging adulthood, including during the transfer from paediatric to adult care.

**Design:**

A qualitative approach was used.

**Methods:**

Individual interviews started in February 2022 and continued to February 2023. Fourteen parents of emerging adults participated. Data were analysed using qualitative content analysis.

**Findings:**

Being a parent of a child with type 1 diabetes involved struggling to accept the loss of control and slowly loosening the ties in the close parent–child relationship during adolescence and emerging adulthood. Parents were gradually reconciling with the illness that was turning the world upside down, making efforts to become a close‐knit family team, relinquishing the priorities of supervision and instead cherishing the relationship, processing that the emerging adult is flying the nest and feeling abruptly cut off by healthcare during the transfer to adult care.

**Conclusion:**

The findings were interpreted as outlining a transitional process among parents that started in the onset period, continuing throughout the life span to their children's emerging adulthood. Parents also transition into new roles along with their children. Parents' experiences of complex daily life, multiple transitions and the abrupt transfer from paediatric to adult care are important to acknowledge.

**Clinical Implications for the Profession:**

Transfer is suggested to be carried out with consideration for the individuals' prerequisites and desires. Parents are important stakeholders guiding the transfer. In adult care, a more family‐centred approach could help parents cope with new challenges in joint clinical visits. Parents should be welcomed to joint clinical visits in adult care with consent and parents could benefit from being offered support from nurses independently.

**Reporting Method:**

The study adhered to EQUATOR guidelines and the COREQ checklist for qualitative studies was used as the reporting method.

**Patient or Public Contribution:**

No patient or public contribution.

## 1 Introduction

Type 1 diabetes (T1D) is the most common lifelong disease in children and adolescents (IDF 2021). Targeted daily acts, such as healthy eating, physical activity, insulin injections, blood glucose monitoring, planning, coping and problem solving, are essential components of the self‐management of T1D (ADCES 2021; de Wit et al. 2022; Lindholm Olinder et al. 2022). If optimal self‐management and treatment outcomes are reached, complications can be prevented or delayed (IDF 2021). This is important since T1D has been known to shorten the life span, especially when diagnosed during childhood (Rawshani et al. 2018). Adolescence and emerging adulthood involve complex environmental, social, behavioural and emotional needs and young people living with T1D are further challenged to achieve and maintain optimal glycaemic outcomes and psychosocial well‐being (Young‐Hyman et al. 2016). Self‐management plans with education and support are recommended, especially in adolescence and emerging adulthood (IDF 2021; de Wit et al. 2022; Lindholm Olinder et al. 2022). Adolescents, emerging adults and parents struggle to meet the demands of the illness when integrating diabetes management in daily life (Young‐Hyman et al. 2016). In diabetes care, parental and family factors are essential for psychological health outcomes during adolescence and emerging adulthood (de Wit et al. 2022).

In this study, transitions are defined as life changes. ‘Emerging adulthood’ is defined as the life period extending from 18 to 29 years (Arnett et al. 2014). ‘Transfer’ is defined as a series of events whereby the person is relocated from paediatric to adult healthcare. In the Swedish diabetes care context, transfer is implemented at the biological age of 18 years, depending on a medical assessment of health status. ‘Parents’ refers to the legal guardian of the child and does not assume the normative pattern of two parents to a child.

## 2 Background

Daily life changes for the entire family when a child is diagnosed with T1D. Adolescence is connected to complex developmental stages, many of which affect blood glucose levels, such as psychosocial, behavioural and hormonal changes (Gregory et al. 2022; Mazur et al. 2017). Meanwhile, these changes occur in the context of developing independence and autonomy concurrently with processing a lifelong illness and natural separation from parents (Gregory et al. 2022; Mazur et al. 2017). Adolescents still need parental support despite increasing independence and autonomy (de Wit et al. 2022; Farthing, Bally, Rennie, et al. 2022; Gregory et al. 2022). Parental support is further needed when dealing with altered healthcare routines during the transfer period from paediatric to adult care (Gregory et al. 2022; Farthing, Bally, Rennie, et al. 2022). However, support from for example extended family members and friends play a crucial role in managing T1D, reducing the burden on parents (Wysocki et al. 2009; Helgeson et al. 2023). As adolescents transition into adulthood, the parent–child relationship evolves significantly. During adolescence, parents often have the responsibility role, managing daily activities and health care routines. However, as adolescents grow into young adults, this dynamic shifts towards a more supportive role where parents begin to encourage greater independence. This shift helps young adults develop self‐management skills and confidence, while still benefiting from their parents' experience and support (Aydın et al. 2024; Berg et al. 2019).

Additionally, demands of diabetes management have changed along with improved knowledge and innovative technologies. Being a parent of an adolescent and emerging adult living with T1D involves complex multifaceted challenges and transitions, as these life episodes are commonly associated with less optimal treatment outcomes (Gregory et al. 2022; International Diabetes Federation [IDF] 2021; Mazur et al. 2017). The International Society for Paediatric and Adolescent Diabetes (ISPAD) recommends assessing general family functioning and the quality of the relationship concerning conflicts and stress. Diabetes‐related family functioning should be discussed, addressing aspects such as communication, support, roles and responsibilities (de Wit et al. 2022). Psychosocial assessments are especially essential when experiencing transitions (de Wit et al. 2022).

The American Diabetes Association (ADA) emphasises that healthcare should promote individual and development‐appropriate family involvement in diabetes management for adolescents and acknowledge that the preterm transfer of responsibility may lead to less optimal self‐management behaviours and treatment outcomes (Young‐Hyman et al. 2016). The importance of parents being involved in diabetes care is highlighted to promote and advocate for a trusting and communicative relationship (Kneck et al. 2023).

Adolescents and emerging adults commonly do not share the same life priorities and views of the illness with parents, leading to family conflicts (de Wit et al. 2022; Farthing, Bally, Rennie, et al. 2022). Parents engaging in realistic goal setting can create a sense of purpose for their adolescents and emerging adults and encourage positive solution‐based thinking (Association of Diabetes Care and Education Specialists [ADCES] 2021). ADA highlights the importance of educating parents in effective problem solving and family conflict management (Young‐Hyman et al. 2016). Parents should therefore be encouraged to develop authoritative and helpful parenting styles with concrete and realistic expectations to promote well‐being (Gregory et al. 2022) and a positive family environment (ADCES 2021).

Parental well‐being and coping strategies affect their emerging adult's health and treatment outcomes (de Wit et al. 2022). Parents of emerging adults living with T1D are exposed to high rates of distress (ADCES 2021; de Wit et al. 2022; Young‐Hyman et al. 2016). Parental coping support is often needed and parenting interventions such as coping skills training have the potential to reduce diabetes distress and fear of hypoglycaemia (de Wit et al. 2022). ISPAD recognises fear of hypoglycaemia during night‐time and its possible but unclear role in death during sleep (called ‘dead in bed’ syndrome) as a source of diabetes distress in parents of emerging adults living with T1D (Abraham et al. 2022). Negative consequences associated with severe hypoglycaemia can increase risks of anxiety, poor sleep and decreased quality of life in parents (Abraham et al. 2022). The parental emotional burden of diabetes (ADCES 2021; Young‐Hyman et al. 2016) and parental stress (Yi‐Frazier et al. 2022) should be emphasised in healthcare. In the diabetes self‐management framework ‘An Effective Model of Diabetes Care and Education: The ADCES7 Self‐Care Behaviours’, healthy coping is placed at the heart of important self‐care behaviours (ADCES 2021). Healthy coping is connected with self‐efficacy and social support and positive attitudes towards diabetes management are associated with improved clinical outcomes (ADCES 2021). Further, The Family Systems Theory emphasises the interconnectedness of family members and how their interactions influence each other's well‐being. For example, in the context of T1D self‐management, parents play a crucial role by providing emotional support and helping with medication adherence (Bowen 1985; Shaw et al. 2021).

ISPAD underlines that diabetes management requires high levels of persistent educational involvement and support, especially during transitions and transfer (Lindholm Olinder et al. 2022). The transfer to adult care should be guided by discussions involving parents and be based on preferences, readiness and regulations (Gregory et al. 2022). It is recommended that healthcare professionals (HCPs) include parents in preparations for the transfer process (Mazur et al. 2017). Parents of emerging adults living with T1D described struggling to find their new role in healthcare after transfer to adult care, letting go of supervision and learning to play a more supportive and counselling role (Pritlove et al. 2020).

This study is relevant since Sweden has the second highest incidence of T1D in children and adolescents in the world (IDF 2021) and despite self‐management interventions, challenges remain in progressing towards improved diabetes‐related outcomes in adolescence and emerging adulthood (Gregory et al. 2022; Lindholm Olinder et al. 2022). Parents' experiences merit study since they may help answer the ‘why’ questions from a psychosocial perspective and further experiences from a country of high incidence is of international importance. Prior studies in this research field have highlighted the experience of parenting children living with T1D during adolescence and emerging adulthood (Goethals et al. 2017; Tomette et al. 2020; Case et al. 2021), but to the best of our knowledge and in our opinion literature regarding the transfer to adult care has mainly described parents' experiences in adolescence, including focus on preparations for the transfer. This study sets out to further explore parents' experiences during their children's emerging adulthood and to describe the experienced transfer to adult care in retrospect.

## 3 The Study

### 3.1 Aim

The aim of this study was to describe experiences of being a parent of a child living with T1D during adolescence and emerging adulthood, including the transfer from paediatric to adult care.

### 3.2 Design

The study applied a qualitative approach and qualitative content analysis was used as the method of analysis. The aim of the study guided our decision on the method of analysis, since we wanted to explore subject and context in describing variations of experiences. We described variations in parental experiences with a focus on exploring manifest and latent content by means of de‐contextualisation and re‐contextualisation (Graneheim et al. 2017; Graneheim and Lundman 2004; Lindgren et al. 2020). To give voice to the parents of emerging adults living with T1D, the data were gathered in individual interviews.

### 3.3 Participants

The participants fulfilled the inclusion criteria of being a parent of an emerging adult (aged 19–29 years) living with T1D who had been transferred from paediatric to adult healthcare at least 1 year ago. The exclusion criterion was whether the child was living with additional long‐term conditions. Fourteen parents, nine mothers and five fathers, participated; nine additional parents were invited but declined. The parents who declined did not reply to our contact efforts, had no interest in sharing experiences, or did not have time to participate. The first eight participants were recruited via convenience sampling in a previous study (Olsson et al. 2023) and the six further participants were recruited via purposeful sampling (via JO) at a university hospital. Parental contact was made only after consent was obtained from the related emerging adult. The parents had experience with their children's diabetes management since childhood or adolescence (range 4–19 years). The emerging adults' diabetes duration before the transfer from paediatric to adult care ranged from 2 to 15 years. The parents were 47–69 years old and lived in rural areas and cities in northern Sweden. Twelve were working and two were retired. Four parents had experience working in healthcare contexts. Seven parents cohabited with their emerging‐adult child.

### 3.4 Data Collection

Before the interviews, parents were informed of the study (by Sara Olsson and Julia Otten) and given opportunities to ask questions before being recruited and giving informed consent. The first author (Sara Olsson) performed and recorded the individual interviews via video link, since visual recording captures non‐verbal cues and contextual information that otherwise might be missed. Acting on the research team's decision, the interviewer (Sara Olsson) informed the participants of her pre‐understanding of the field as living with T1D herself. Data collection started in February 2022 and continued until February 2023. An interview guide containing open‐ended and probing questions was used (Table 1). The opening item was ‘Please, tell me about when your child got diagnosed with T1D’. The interviews lasted 21–66 min and were transcribed verbatim (by Sara Olsson). We anticipated that we had collected enough data to describe significant variations of experiences (cf. Graneheim et al. 2017) by the twelfth interview, but performed two additional interviews to see whether further data would improve our understanding. It was determined that such additional data did not contribute to a better comprehension of the matter.

**TABLE 1:** The semi‐structured interview guide.

Question areas	Questions
Life with T1D throughout the life span	Please, tell me about when your child got diagnosed with T1D. When, where, how? Experiences, memories, emotions, etc. Tell me about your life as a parent of a child with T1D today. Tell about your views of the illness. Advantages, disadvantage and challenges. Meanings and consequences regarding school, education, friends, spare time, activities, etc. How has life with T1D affected your family and your own relationships?
Development of independence	How does your child handle self‐managing the illness with everything that is involved? When did you first experience the self‐management as your child's own responsibility? What is your role in diabetes management?
Paediatric care	Tell me about your experiences at the paediatric clinic. How do you experience the support from healthcare professionals in paediatric care? Tell me about your expectations of the adult clinic.
Transfer and adult care	Tell me about your experiences of the transfer to adult care. Timing, practical aspects, emotional aspects, care relations, etc. How would you describe your experiences of being a parent of an emerging adult who is currently living with the responsibility to self‐manage T1D? What challenges has your child experienced during emerging adulthood? Tell me about your experiences of the support from healthcare professionals at the adult clinic and other possible sources of support. If you had the opportunity to affect how the transfer from paediatric to adult care should be carried out, how would you like it to be?
The future	What opportunities do you see for your and your child's future? Use and interest of supportive technology for diabetes management, contacts with healthcare, etc.
Probing questions	What does that mean to you? How did that make you feel? How did that affect you?

### 3.5 Data Analysis

Data were analysed using qualitative content analysis with an inductive approach, with the content being abstracted and interpreted according to Graneheim et al. (2017), Graneheim and Lundman (2004) and Lindgren et al. (2020). The analysis was performed collaboratively by Sara Olsson, Åsa Hörnsten and Madeleine Blusi. To begin the process during the de‐contextualisation phase, a general overview was obtained by reading the transcribed text several times before dividing it into meaning units. Meaning units were condensed to make them shorter without losing their core content and were coded based on content. During the re‐contextualisation phase, codes were contrasted and organised into groups based on similarities and differences to create sub‐themes, which were further abstracted into themes. Finally, a main theme was interpreted that covered all relevant data. The process of qualitative content analysis was dynamic and entailed interactive movement between the transcribed texts, code sorting, abstraction and interpreting themes at various levels. Notably, we reflected upon our pre‐understandings of diabetes during the entire analytical process. The reframing and making sense of data is mutual as co‐creations are emphasised in qualitative content analysis (Lindgren et al. 2020) and no descriptions are free from interpretations (Sandelowski 2000 ). Finally, after adjustments and discussions, the findings were processed and approved by all members of the research team.

### 3.6 Ethical Considerations

Ethical approval (Dno 2021‐02873) was obtained 20 June 2021 from the Swedish Ethical Review Authority. In accordance with the ethical guidelines of the Helsinki Declaration and the World Medical Association (WMA 2022), the participants were informed of the purpose of the study, of conceivable risks and benefits and of efforts made to maintain health, integrity and confidentiality. They were also informed of their prerogative to determine their participation, which was voluntary. One conceivable risk was that the interviews could cause emotional discomfort, so the study information letter provided contact information for independent consultation if further emotional support were desired.

### 3.7 Trustworthiness

Although we could not determine a preferred number of participants beforehand (Graneheim et al. 2017), we believe that we gathered sufficient data from participants of various ages and genders to capture considerable variations in experiences. The last two interviews, with the associated codes, did not result in new subthemes, indicating data saturation. By adding interviews and codes without new emerging subthemes, we believe that we have captured meanings of the experiences in display related to the subject and context and therefore closed the recruitment process. Six participants were recruited via purposeful sampling based on care context as their contact with the responsible physicians in adult care ensured relevant experiences. Several research team members have personal experiences with diabetes, which were discussed beforehand to manage pre‐understandings. While this came with benefits and risks, we believe our findings capture the most probable meanings of the parental perspectives elicited. Over‐interpretation was mitigated through discussions. During de‐contextualisation, codes were kept close to the transcribed texts (cf. Lindgren et al. 2020) and rare experiences were coded and supported with quotations. We carefully described the participants, including the participants who declined and procedures, allowing readers to determine the transferability of the findings to other contexts (Graneheim and Lundman 2004). The first author (Sara Olsson) evaluated the study reporting using the 32‐item COREQ checklist for qualitative studies with in‐depth interviews (Tong et al. 2007) (see Appendix 1).

## 4 Findings

The findings of this study are organised in terms of a main theme, five related themes and 15 subthemes. Overall, the findings (Table 2) showed that experiences of being a parent of a child living with T1D during adolescence and emerging adulthood involved struggling to accept the loss of control and slowly loosening the ties in the close parent–child relationship.

**TABLE 2:** Findings presented as main theme, themes and subthemes.

Main theme	Themes	Subthemes
Struggling to accept the loss of control and slowly loosening the ties in the close parent–child relationship	Gradually reconciling with the illness that was turning the world upside down	Wondering why this happened ‘Normalising’ family life and re‐evaluating the situation over time
	Making efforts to become a close‐knit family team	Trying to be sufficient for all family members Acting strong when somebody else feels weak Sharing and comparing experiences with others
	Relinquishing the priorities of supervision in disease management and instead cherishing the relationship	Striving to be good parents during emotional storms Continually on guard despite revolutionary supportive technology Learning to respect the emerging adults' integrity Understanding that pleasures of emerging adulthood outweigh demands of the disease
	Processing that the emerging adult is flying the nest	Reconciling with the adolescent's own choice of lifestyle Struggling to stand back and hand over the responsibility Making sure that the emerging adult get social support in community networks
	Feeling abruptly cut off by healthcare during the transfer to adult care	Missing long‐term caring relations with HCPs Losing personal impact after being included and involved Wanting healthcare to consider the emerging adult's individual conditions

### 4.1 Gradually Reconciling With the Illness That Was Turning the World Upside Down

The experience of being a parent of a child living with T1D during adolescence and emerging adulthood involved a process of gradually reconciling with the illness that was turning the world upside down. The onset period involved *wondering why this happened, ‘normalising’ family life and re‐evaluating the situation over time*.

#### 4.1.1 Wondering Why This Happened

Parents struggled to cope with feelings of despair and frustration as to why this had happened to them, particularly when they did not have any prior experiences of T1D in relatives:

In our wildest imagination, we couldn't believe that this would happen to him or our family. It doesn't have to be genetic, but they say it mainly is … and someone has to be the first. (Participant 13)

The parents emphasised that when something as serious as this happens in your family, you are deeply moved, even if you have previous experience of T1D or healthcare. A parent who had lived with T1D had regularly checked the child's blood glucose at home, perhaps to catch the disease at an early stage if the child was going to get diagnosed too—a fear that was realised 1 day.

Parents either remembered clearly or had only a foggy recollection of the exact course of events when their child was diagnosed. The period of T1D onset involved existential trials and some felt helpless and scared. They wanted to lighten the burden for the child, yet this was in conflict with the need to motivate the child to adhere to new demands for health.

#### 4.1.2 ‘Normalising’ Family and Re‐Evaluating the Situation Over Time

A large shift in family life was initiated and required because of the diagnosis, but one parent expressed the importance of efforts to keep family life as ‘normal’ as possible when integrating disease management, such as by viewing T1D as a friend and not a rival. Family life was reformed and, for example, participation in dinner parties and vacations was stopped due to lack of energy. Coordinating diabetes management became the centre of family life. The onset period was reportedly an undramatic transition in a family in which diabetes management was already integrated, but it was still absurd to have an additional family member living with T1D. Parents said that the diabetes forced them to plan a more structured family life, which was beneficial for everyone, although they would rather not have had to deal with diabetes. Parents gradually reconciled with and re‐evaluated the situation over time:

There's a huge difference in being her parent now compared with when she was a teenager. We went through this journey together and landed. In the past, it was chaotic and terribly tiresome. Day and night, it was always on my mind, but now we don't—we're just living instead. (Participant 2)

In retrospect, parents reflected on some positive consequences of learning to live with diabetes management. Some believed that T1D is simply a condition and that, years later, diabetes management was a natural part of family life; some did not even remember what it was like living without it.

### 4.2 Making Efforts to Become A Close‐Knit Family Team

Being a parent of a child living with T1D during adolescence and emerging adulthood involved making efforts to become a close‐knit family team and parents were *trying to be sufficient for all family members by acting strong when somebody else feels weak. Parents also reported the importance of sharing and comparing experiences with others*.

#### 4.2.1 Trying TO Be Sufficient for All Family Members

Parents spoke of self‐criticism and felt guilty regarding their efforts to be sufficient for all family members. A parent speculated that in every family with children there is some kind of disease, either physical or psychological, so every family has its own problems to deal with; believing that one can always be sufficient for everyone and always maintain well‐being is actually not realistic. Parents with several children reported that even though they were concerned about all their children, they still perceived greater concern for the child living with T1D:

How's it for siblings of children having lifelong diseases? Our daughter has been characterised by stepping aside for her sister and has made allowances for her disease management and we [the parents] have regarded her type 1 diabetes endlessly. (Participant 10)

A parent gained retrospective insights from a sibling's view of the child living with T1D as being spoiled. Parents reported siblings being irritated at the greater attention paid to the child living with T1D, explained by the parents as the siblings not fully understanding the severity of T1D and the effort it required.

#### 4.2.2 Acting Strong When Somebody Else Feels Weak

Becoming a close‐knit family team by acting strong when someone else felt weak, with complementary efforts, led to relief to be sharing the burdens with a co‐parent. All parents lived with a co‐parent with whom they had a relationship when the child was diagnosed with T1D, but three participants had since divorced. Parents felt obliged to act strong when someone else in the family was weak:

Well, [i.e., the father] became depressed and I believe that if someone is weak the other one has to act strong and try to keep up the good spirits. (Participant 7)

Parenting a child living with T1D involved constant worries and anxiety and parents displayed different coping strategies. Some parents never tried to hide their emotions and their children were aware of the parental diabetes burden. Others kept their worries to themselves so as not to let the worries affect their children. The situation of playing it cool and portraying oneself as content and happy, while actually being scared to death, was described. A parent expressed feelings of being on the run inside. Both permanent and temporary sickness leave was reported. Diabetes management and the processing of experiences had affected the parents' own relationships and parents described their own health as vulnerable:

You see, everything tangles up completely when stuff like this happens. You're fragile, the smallest things can cause chaos and I need to stay healthy too … When you have children, you always put them first and yourself last. (Participant 2)

#### 4.2.3 Sharing and Comparing Experiences With Others

Parents described the importance of sharing experiences in community with others. Comparing experiences might also be empowering, revealing that your family seemed to have integrated strategies better than other families. In addition, a parent believed that sharing experiences of ‘normal’ emerging adult behaviour was crucial too. Some parents had shared experiences in a parental support group in paediatric care, while others had not wanted to. The impact of parental support groups was not always positive:

We attended, but it was hard since we didn't live there [i.e., in the city where the paediatric clinic was located]. We didn't have any contacts with other parents … That's why it felt a bit strange when we went there, we felt left out because we didn't know anyone. And well, maybe that's why we got divorced—we couldn't take it any longer. I don't know, it's possible. That thought has crossed my mind sometimes. (Participant 7)

In adult care, several parents believed that peer support was meaningful for the emerging adults, but not for them.

### 4.3 Relinquishing the Priorities of Supervision in Disease Management and Instead Cherishing the Relationship

Being a parent of a child living with T1D during adolescence and emerging adulthood involved relinquishing the priorities of supervision in disease management and instead cherishing the relationship. The parents *were striving to be good parents during emotional storms. They were continually on guard despite revolutionary supportive technology, learning to respect the emerging adults' integrity and understanding that the pleasures of emerging adulthood outweigh the demands of the disease*.

#### 4.3.1 Striving TO Be Good Parents During Emotional Storms

Some parents had almost daily conversations with their emerging‐adult children about the emotional burden of T1D, while others stated that it was difficult to speak about such emotional issues. They argued that it was up to the emerging adult to initiate discussions of emotional burdens. Parents reported ups and downs during their children's adolescence and emerging adulthood and they were striving to be good parents during emotional storms:

To be in puberty [and learning to live with T1D]—it was very hard on the relationship, it was difficult to be a good parent when many things were happening concurrently. … Her being extremely frustrated and to stand there as a parent and comfort her—that's been really difficult, since it's actually extremely hard for her. (Participant 8)

#### 4.3.2 Continually on Guard Despite Revolutionary Supportive Technology

Parents were helped to relinquish the priorities of supervision by revolutionary supportive technology for diabetes management. Despite these technological advances, bolstering safety and control, parents were still continually on guard. However, some trusted humans more than technological devices. Parents were particularly emphatic as to the importance of getting personal feedback from their children in addition to shared blood glucose statistics:

Sometimes his father had already sent him a text. He [i.e., the son] found it terribly tiresome when we sent him multiple messages and that's how we decided to always contact him only on Messenger to be able to see each other's messages. (Participant 5)

Several parents wanted to be connected to the continuous glucose monitor (CGM) applications that let them follow their children's blood glucose levels on their smartphones, but were no longer allowed to do so by their emerging‐adult children. In one case, a parent was still connected to the CGM and the emerging adult encouraged the parent to contact her when needed. In another case, the smart applications with alarms caused overwhelming emotional responses due to a previous ketoacidosis episode:

All systems get triggered … I don't want it [i.e., the smart application for CGM] since it becomes too stressful for me. (Participant 11)

#### 4.3.3 Learning to Respect the Emerging Adults' Integrity

Emerging‐adult children demanded their integrity and their parents had to learn to respect that. A parent wondered whether the emerging adult appreciated the parental T1D‐related questions, or whether he returned feedback only for the parent's sake. A balancing act between checking in with loving concern and being intrusive and nagging was reported: if one pushes it too far, one might end up losing the close connection. In situations in which parents perceived a need to contact the diabetes team related to the emerging adult's mental health, they still did not due to integrity concerns. Several parents also used global positioning systems (GPS) on their smartphones to see where their emerging‐adult children were. However, one emerging adult asserted his independence in this matter:

We're used to being able to see the blood sugar levels in our phones, but he disconnected us. He had allowed us to be friends with him on SnapChat to be able to see where he is. Fortunately, sometimes he allows us to see where he is and sometimes not. … we pleaded, ‘If you're going to a party tonight, please allow us to see’. … But, no. (Participant 5)

#### 4.3.4 Understanding That Pleasures of Emerging Adulthood Outweigh Demands of the Disease

Understanding that the pleasures of emerging adulthood outweigh the demands of the disease was part of the process of relinquishing the supervision of disease management and instead cherishing the relationship with the emerging‐adult child beyond T1D. A parent expressed the importance of general life discussions:

She tells me things and then we talk about it [i.e., diabetes], otherwise we don't. She lives her own life and that's what we talk about when we meet. … Because we have a relationship beyond the disease too and we need to cherish it. (Participant 8)

Some parents reported that they had fully let go of the T1D supervision, while others still had difficulties cherishing the new relationship dynamics. Their understanding that the pleasures and general life outweigh the demands of the disease surfaced when the emerging adults were travelling:

I need to get better at promoting fun first and later talking about how to do the packing. For me it's a great horror when a group of 19‐year‐old boys goes travelling—I know what that means, they are not going to visit museums. … But when I see that the travelling works out fine, I believe that nothing's impossible. (Participant 5)

### 4.4 Processing That the Emerging Adult Is Flying the Nest

Being a parent of a child living with T1D in adolescence and emerging adulthood involved processing that the emerging adults were flying the nest. Parents were *reconciling with the adolescent's own choice of lifestyle* and were *struggling to stand back and hand over the responsibility*. Parents emphasised *making sure that their children got social support in community networks*.

#### 4.4.1 Reconciling With the Adolescent's Own Choice of Lifestyle

Parental processing that their emerging‐adult children were flying the nest was related to decreased parental responsibility for diabetes self‐management, as the adolescent children were becoming more independent in their lifestyle choices. Parents argued that it is important to teach their children that T1D should not be seen as a source of limitations in life. A parent described the struggle within the emerging adult in periods when it was clear that T1D in fact was a limitation and this was a great source of worry for the parent. Some parents felt conflicted about not viewing T1D as a source of limitation since the emerging adult was perceived as taking too many risks:

She's been very clear that ‘I can do anything and this [i.e., T1D] will not stop me’—it became like a mantra. Sometimes, it felt almost reckless. … Life consists of many restrictions. I can't do exactly everything I want either, due to who I am. … But, you could absolutely not say that to her. (Participant 8)

Adolescence and emerging adulthood were characterised by de‐prioritised self‐management. Emerging adults' own lifestyle choices included new challenges such as drinking alcohol—a great parental concern. Parents could not restrict them from drinking but asked for transparency about their efforts to be prepared.

#### 4.4.2 Struggling to Stand Back and Hand Over the Responsibility

Parents struggled to take a step back in diabetes management and trust their adolescent children with responsibility. They reported a process of reduced involvement. HCPs had a major role in engaging with adolescents to develop their independence, which in turn engaged parents in the importance of learning to stand back:

They [i.e., in paediatric care] had told him that perhaps the parents need to back down too, consequently he was also pushing us. … I believe that this benefited him, he's been empowered that the responsibility is his. I thought I would never be able to … let him do anything on his own. It's a process. (Participant 5)

In contrast, some never struggled to stand back and hand over responsibility due to never hesitating over the emerging adult's independent capabilities. Some maintained that their child was not taking command of self‐management despite being an emerging adult. Struggles over stepping back and handing over responsibility became clear when emerging‐adult children moved away from home. One parent commented on the struggle:

You want to follow them as a shadow and help them out. Still, I guess you can't help them too much either. They need to be able to grow as individuals, you can't be overprotective. The most important thing is to make sure they feel that they can always turn to you if they feel insecure. … You need to let them make their mistakes. (Participant 3)

#### 4.4.3 Making Sure That the Emerging Adult Get Social Support in Community Networks

Parents felt safe when their emerging‐adult children kept spending time with old friends who were familiar with diabetes management and they wanted to make sure that their children got social support in community networks. Besides, the parents knew these friends and their parents. Related to emerging adults starting to fly the nest, parents felt safe when their emerging‐adult child was living with a partner. The parents expressed concerns when their emerging adults started university studies. A parent raised a concern about the emerging adult in new social environments:

When you're in school, all your friends know that you have diabetes. But later when you're an adult and in new contexts, not everybody knows. And perhaps you don't want all of them to know, you don't want to be the one with diabetes. In the first place, you just want to be you. (Participant 4)

### 4.5 Feeling Abruptly Cut Off by Healthcare During the Transfer to Adult Care

Being a parent of a child living with T1D during adolescence and emerging adulthood involved feeling abruptly cut off by healthcare during the transfer to adult care. Parents were cut off *and missing long‐term caring relations with HCPs* and were *losing personal impact after being included and involved*. Parents *wanted healthcare to consider the emerging adults' individual conditions* during the transfer.

#### 4.5.1 Missing Long‐Term Caring Relations With HCPS

Parents felt cut off from long‐term caring relations with HCPs during the transfer. Paediatric nurse specialists were described admiringly and were even available at night‐time. Continuity and long‐term caring relations provided security.

All emerging adults were transferred at 18 years old, as the Swedish healthcare system mandates. In some cases, a paediatric nurse specialist followed the emerging adult and a parent to adult care. In other cases, the emerging adult transferred to the adult clinic and met the new diabetes team alone. Parents expressed a desire to meet these new HCPs:

If you'd had a transfer period at the paediatric clinic with the diabetes nurse specialist from adult care, you would have the opportunity to start to create caring relations. You simply have them as names on a piece of paper stuck to the fridge with phone numbers. (Participant 9)

Parents desired a bridge between paediatric and adult care, instead of the described gap. They discussed the possibility of including adult care HCPs in paediatric care before the upcoming transfer. A few parents had personal caring relations with HCPs at the adult clinic, but most of them did not.

#### 4.5.2 Losing Personal Impact After Being Included and Involved

Parental involvement had gradually decreased and a total loss of personal impact was felt in adult care: they were cut off. A parent reported almost crying when leaving paediatric care, while the emerging‐adult child was ready to start over at the adult clinic totally independently. The parent and child did not share the same experience of the transfer.

Parents believed that HCPs from adult care suggested that the emerging adult should anticipate complete independence. The sense that parents were not allowed to participate in their children's adult care came from both the emerging‐adult children and healthcare. Some parents were allowed by the emerging adult to participate in the first visit, due to the parent's desire. A parent commented on a perceived difference between communication in paediatric and adult care:

I followed her on the first visit [to the adult clinic]. … I remember being ignored, like I wasn't in the room. … From being 100% involved … I was with her almost every visit to the paediatric clinic, all three of us talked together about how my daughter was doing and how it was at home. I had a bird's eye view. … It concerned us all there and suddenly I was treated like air. (Participant 11)

Some parents wanted to accompany their emerging‐adult child on their first visit to adult care but were conflicted about doing so, since they thought that this would elicit negative reactions from HCPs or because they themselves viewed doing so as a failure. Parents asked themselves whether the desire to be in control arose within themselves, or whether they deemed their child to actually require help with diabetes management during emerging adulthood.

#### 4.5.3 Wanting Healthcare to Consider the Emerging Adult's Individual Conditions

Parents wanted consideration of the emerging adult child's individual preferences and conditions concerning the transfer to adult care. They discussed the importance of respecting individual prerequisites:

Parents or children, all are unique. It has to do with individual conditions. … It's hard when it has to do with emotions and personality. You can never say what is right or wrong, it's case by case. (Participant 3)

Some said that the age of 18 years was ideal for the transfer since it is the age of majority and most adolescent children were still living with their parents. The age of majority was perceived as a sign to let go, although this was perceived as a struggle, while others stated that a fixed age was problematic, referring to individual conditions. Instead, some parents suggested that the transfer should be guided by diabetes duration.

## 5 Discussion

Five themes were identified describing the experiences of being a parent of a child living with T1D during adolescence and emerging adulthood, including the transfer from paediatric to adult care in retrospect: gradually reconciling with the illness that was turning the world upside down, becoming a close‐knit family team, relinquishing the priorities of supervision and instead cherishing the relationship, processing that the emerging adult was flying the nest and feeling abruptly cut off by healthcare during the transfer to adult care. These themes were interpreted as outlining a possible transitional process in parents that had already started in the onset period, continuing throughout the child's early life and into emerging adulthood. The process involved managing a complex daily life and multiple transitions with an abrupt transfer to adult care. Parents were struggling to accept the loss of control and were slowly loosening their ties in the close parent–child relationship. The findings were elaborated in relation to Transitions theory developed by Meleis (2010). Schumacher and Meleis (2010) described four types of transitions: developmental, situational, health‐illness and organisational. In relation to the possible outlined transitional process in parents, all four types of transitions are involved simultaneously, adding vulnerability. Developmental transitions such as during adolescence and emerging adulthood, situational transitions in educational roles involving, for example, T1D‐related consequences of alcohol‐drinking, health‐illness transitions as in living with a life‐long disease and experiencing different levels of care and finally, organisational transitions in being transferred to adult care where the parents need to adapt to new practices. Importantly, nurses are recommended to support meanings, expectations, preparations and planning, knowledge levels, environments, as well as emotional and physical well‐being attached to the transitions (Schumacer and Meleis 2010).

The findings related to gradually reconciling with the illness that was turning the world upside down emphasised that experiences of being a parent of a child living with T1D involved existential trials in the onset period. This study focused on parents' experiences of their child's adolescence and emerging adulthood, but parents still frequently referred to the onset period, since having their child diagnosed was said to be the start of a series of life‐changing transitions. Several studies have reported that parents need to learn to accept a new reality when integrating their child's T1D (de Wit et al. 2022; Holmström Rising and Söderberg 2023; Pritlove et al. 2020). Parents in this study sensed a decline in their child's independence, for example, related to the supervision of blood glucose levels at night and other studies have reported this as parents being fully occupied with processing their child's disease at all hours (Holmström Rising and Söderberg 2023; Yi‐Frazier et al. 2022).

Many studies indicate that all family members are affected when a child is diagnosed with T1D (Carlsund and Söderberg 2018; de Wit et al. 2022; Gregory et al. 2022; Yi‐Frazier et al. 2022). In this study, becoming a close‐knit family team was considered important when adjusting to the demands of the disease. Parents in this study set aside their own health when they tried to be sufficient for other family members. Becoming a close‐knit family team involved dealing with emotional burdens. Diabetes distress is common in parents and adolescents living with T1D (Young‐Hyman et al. 2016). Castensøe‐Seidenfaden et al. (2016) explored adolescents' and parents' perspectives on living with T1D and the dyad shared the same concerns and challenges, but usually hesitated to share their emotions in efforts to protect each other. In our study parents were ambivalent in sharing their emotions related to diabetes distress with their emerging adults. The literature highlights the importance of supporting all members of the family team (ADCES 2021; Carlsund and Söderberg 2018; de Wit et al. 2022; Gregory et al. 2022; Holmström Rising and Söderberg 2023; Holtz et al. 2020; Lindholm Olinder et al. 2022; Pritlove et al. 2020; Yi‐Frazier et al. 2022). The findings revealed that acting strong, despite experiencing vulnerability, could lead to exhaustion syndrome and sickness leave. Ignoring one's vulnerability and putting on a front have been described in a previous study of mothers of children living with T1D experiencing exhaustion syndrome (Lindström et al. 2017). Holmström Rising and Söderberg (2023) reported likewise that diabetes management can interfere with work performance and that T1D may affect an entire work career, leading to affected family finances. Parents highlighted the importance of sharing experiences in community and today peer support through social media is a valuable source of counselling (Carlsund and Söderberg 2018; Gregory et al. 2022; Holmström Rising and Söderberg 2023). Related to becoming a close‐knit family team living with T1D and diabetes management, it is emphasised to recognise other important caregivers or extended family, such as grandparents (Carlsund et al. 2026), partners (Carlsund and Söderberg 2018) and peers (Helgeson et al. 2023). In addition, it is necessary to let the emerging adults define who qualifies as ‘family’ (Zysberg and Lang 2015), since the children naturally becomes more distant from parents during emerging adulthood.

The findings indicated that parents were learning to relinquish the priorities of supervision in disease management and instead cherishing the relationship with their emerging‐adult children and they described themselves as being continually on guard despite revolutionary supportive technologies. These qualitative findings about parental experience supplement the rapidly expanding field of supportive technology for diabetes management. HCPs are challenged to keep up with the growth of technology and learn its complexities (ADCES 2021; Lindholm Olinder et al. 2022). Parents still emphasised the relational aspect of direct personal feedback when they monitored blood glucose and statistics via CGM. Human input is still required to identify appropriate applications and tools, to educate oneself and to analyse and facilitate the use of the supportive technologies and the information they can offer (ADCES 2021). ISPAD highlights that HCPs need to provide education and support that increases family members' knowledge and understanding of supportive technology, while concurrently helping them to learn about the impact on daily life (Lindholm Olinder et al. 2022). In our study parents had been thoroughly introduced to how the support technology for diabetes management should be used, but the parents did not mention discussions on its emotional impact on daily life. Perhaps HCPs ought to further focus on the relational factors in using supportive technology. Related to aspects of cherishing the relationship beyond T1D, Yi‐Frazier et al. (2022) suggested that attention should be paid to helping parents navigate relationships with their children during their adolescence and emerging adulthood. ISPAD (Lindholm Olinder et al. 2022) reviewed parents' obligations to combine diabetes management duties with fundamental parental responsibilities, which in our study was demonstrated to be challenging. These findings indicate two paths to cherishing the parent–child relationship: learning to respect the emerging adult's integrity and understanding that the pleasures of emerging adulthood sometimes outweigh the demands of the disease.

In addition, processes of reconciling with the adolescents' and emerging adults' own lifestyle choices were reported when processing that the emerging adult is flying the nest. A prominent parental concern about the adolescent's lifestyle choices concerns alcohol drinking. Yi‐Frazier et al. (2022) described developmental milestones contributing to parental stress concerning emerging‐adult children living with T1D, such as risk‐taking behaviour involving alcohol drinking when moving from home. Some of the parental issues identified in our findings related specifically to the struggle to stand back and hand over the responsibility for self‐management. The potential desire for even stricter parental involvement due to common suboptimal treatment outcomes enhances parental struggles to let go of responsibilities in diabetes management and their expertise in healthcare (Pritlove et al. 2020). Farthing, Bally, Rennie, et al. (2022) reviewed the division of T1D management responsibilities between adolescents and their parents and concluded that diabetes nurse specialists should observe and ensure the integration of shared responsibilities. Parents in our study were struggling to accept the loss of control and slowly loosening their ties in the close parent–child relationship, which can be related to the complex process of interdependence described by Farthing, Bally, Leurer, et al. (2022). Interdependence can be described by parents and adolescents living with T1D as teamwork and developing support networks in disease management (Farthing, Bally, Leurer, et al. 2022).

Parents in this study reacted to feeling abruptly cut off by healthcare when their emerging adult child was transferred to adult care. Parents lost their involvement and personal impact in healthcare. It is recommended that active parental involvement in healthcare continue from paediatric to adult care in a transfer process (de Wit et al. 2022; Mazur et al. 2017). Parents have emphasised that the expectation that emerging adults should be totally independent at 18 years of age, when transferred to adult care, is perhaps not realistic (Pritlove et al. 2020). In addition, it is perhaps not realistic that healthcare should assume that parents have learned to live with their new role in diabetes management when transferring their children. Person‐ and family‐centred communication in healthcare focuses on young people living with T1D and their families by promoting their perspectives, with the aim of optimising self‐management and treatment outcomes in the context of the unique person living with T1D (de Wit et al. 2022). Pritlove et al. (2020) reported that parents experienced a healthcare approach in which HCPs sometimes assume a one‐size‐fits‐all transfer process and our findings confirm that the transfer process should be individualised. ADA also recommends initiating discussions of the transfer at least 1 year prior to the start of the process and including the parents in these discussions (Young‐Hyman et al. 2016). In this study, parents suggested that the period of transfer could be guided by diabetes duration. Diabetes duration can help build situational awareness of the process of achieving independence in self‐management and in the healthcare system when initiating the transfer process. In adult care, ethical consideration should be given to the family's perspective to benefit all people involved (Carlsund and Söderberg 2018). The European Academy of Paediatric consensus statement on the successful transition from paediatric to adult care for adolescents with lifelong disease (Mazur et al. 2017) suggested five components of a successful transfer: paediatric and adult‐care HCPs involved according to a written structured policy; personal and parental involvement in transfer preparations and planning; access to multidisciplinary HCP teams; acknowledgement of the young person's needs; and continuity in care, collaboration, as well as financing.

### 5.1 Limitations

The pre‐understandings were comprehensive due to personal experiences of diabetes in the research team. However, these pre‐understandings were handled with care in discussions during the research process to avoid over‐interpretation and to recognise whose voices were being presented (cf. Graneheim et al. 2017). The benefits of having an interviewer living with T1D (SO) included the opportunity to deepen the conversations. Some participants asked the interviewer questions about her parents' experiences, which indicated that alliances were being made and power relations avoided to create open dialogue. Related to the sampling methods used, the convenience sampling described by Elo et al. (2014), was valuable as we had no direct contact with parents. The parents invited via convenience sampling were recruited because they fulfilled the inclusion criteria and had experience in the subject and context. After the transfer to adult care, parents are no longer automatically included in the diabetes care since the emerging adults living with T1D are safeguarded with confidentiality and secrecy. Importantly, we had to ask them for consent and initiative to provide us with contact information to their parents, which can be elaborated as a limitation. The purposeful sampling method described by Sandelowski (2000), was used to invite parents via emerging adults living with T1D at an adult clinic, who fulfilled the inclusion criteria and had an interest in sharing their experiences of the study aim. A limitation concerned the lack of variation in the participants' cultural backgrounds, with only one of the parents having been born in a country other than Sweden. Female participants were over‐represented. However, we did not intend to compare or contrast mothers' and fathers' experiences during the process of analysis, so differences between genders are not presented. The nine invited parents who declined participation might have added more variation in the cultural backgrounds. After recruitment, it was found by chance that four of the parents had experience of working in healthcare contexts, which most probably influenced their experiences in the studied phenomena. However, we believe that this added to the variations of experiences. In two of the interviews, the interviewer noticed that the emerging‐adult child was present in the same room, which could have affected the parents' abilities to deepen the conversations, but these parents still shared their difficulties and emotions. We need to trust the participants in their choice of where they felt most comfortable performing the interview. In view of this, these two participants were nevertheless included in the study. In a larger health perspective, the impact of the COVID‐19 pandemic may have had an impact since some emerging adults were transferred from paediatric to adult care, as well as the data collection, in years under the influence. It is a possible limitation that we did not address this aspect. Furthermore, the emerging‐adult children of the participants ranged from 19 to 29 years of age, so their transfers varied from being recent to longer‐standing. We did not state or compare parental experiences dependent on the emerging adults' ages or their diabetes duration before the transfer, as this was not the aim of this study. The wide age distribution of the participants' children led to full coverage of the period of emerging adulthood. Being transferred longer ago was naturally connected with not remembering all the facts concerning the transfer under discussion. Overall, the study mainly focused on being a parent of an emerging adult living with T1D, which also included the transfer to adult care.

## 6 Conclusions

Along with the children living with T1D, parents also transition into new roles, struggling to accept the loss of control and slowly loosening the ties in the close parent–child relationship during adolescence and emerging adulthood. The current data highlight the importance of acknowledging parents' experiences of complex daily life, multiple transitions and abrupt transfer from paediatric to adult care, since parents' experiences and health affect their children. If healthcare supported parents in their children's transitions, perhaps parents would be better able to empower their emerging‐adult children. Related to the abrupt transfer to adult care, parental experiences should be considered due to the complex nature of self‐management. Transfer to adult care should be considered a transitional process, a period with a series of events rather than being viewed as one event. Diabetes nurse specialists should not view the transfer process as completed when the emerging adult is transferred to adult care. It is suggested that transfer be carried out with consideration for the individuals' prerequisites and desires and parents are important stakeholders guiding the process. In adult care, perhaps a more family‐centred approach could help parents and emerging adults better cope with new T1D‐related challenges. If the emerging adult consents to participation, parents should be welcomed to joint clinical visits in adult care and be offered support from nurses in their transition into new roles. Parents could benefit from being offered support from nurses independently, despite having emerging‐adult children.

Several studies are exploring experiences of being a parent of a child living with T1D during adolescence and emerging adulthood with a focus on preparing for the upcoming transfer from paediatric care. Despite the lessons to be gained from these valuable experiences, questions remain as to how to support and integrate parents in the adult care of their children. Additional research is needed to better understand the challenges in the period after the transfer, challenges experienced by all stakeholders, such as emerging adults, their parents and HCPs.

## Author Contributions

All authors approved the final version of the manuscript for publication and made substantial contributions to design, analysis, or interpretation of the data. All authors critically drafted and revised the work to ensure its intellectual content and furthermore, all agreed to be accountable to all aspects of the work.

## Funding

Funding has been received by The Swedish Diabetes Foundation.

## Ethics Statement

Ethical approval (Dnr 2021‐02873) was obtained 20 June 2021 from the Swedish Ethical Review Authority.

## Conflicts of Interest

The authors declare no conflicts of interest.

## Data Availability

The data that support the findings of this study are available on request from the corresponding author. The data are not publicly available due to privacy or ethical restrictions.

## Appendix 1 Consolidated Criteria for Reporting Qualitative Research (COREQ): A 32‐Item Checklist (Tong et al. 2007)

**Table:**

No.	Item	Guide questions/description	Text reference
Domain 1: Research team and reflexivity			
Personal characteristics			
1.	Interviewer/facilitator	Which author/s conducted the interviews or focus group? Sara Olsson.	3.4 Data collection
2.	Credentials	What were the researcher's credentials? Sara Olsson, R.N, paediatric nurse specialist, PhD‐student. Åsa Carlsund, R.N, district nurse specialist, PhD, lecturer. Madeleine Blusi, R.N, PhD, lecturer. Julia Otten, M.D., PhD, lecturer, Endocrinologist. Elena Lundberg, M.D., paediatric endocrinologist. Ingela Lavin, R.N, diabetes nurse specialist, paediatric nurse specialist. Åsa Hörnsten, R.N, district nurse specialist, professor.	Title page
3.	Occupation	What was their occupation at the time of the study? See box 2.	Title page, 3.3 Participants
4.	Gender	Was the researcher male or female? All members of the research team are female.	Title page
5.	Experience and training	What experience or training did the researcher have? The interviewer (SO) had previous training and feedback from a prior interview study.	3.4 Data collection
Relationship with participants			
6.	Relationship established	Was a relationship established prior to study commencement? The interviewer had no relationships established with the participants.	3.3 Participants, 3.4 Data collection
7.	Participant knowledge of the interviewer	What did the participants know about the researcher? e.g., *personal goals, reasons for doing the research*. The interviewer's (SO) occupation as PhD‐student and paediatric nurse specialist, the research project where this study is included, age, city of residence and the pre‐understanding of living with type 1 diabetes herself.	3.4 Data collection, 3.7 Trustworthiness
8.	Interviewer characteristics	What characteristics were reported about the interviewer/facilitator? E.g., *pre‐understanding, assumptions, reasons and interests in the research topic*. The interviewers pre‐understanding is reported together with the pre‐understandings of all the research team members.	3.4 Data collection, 3.7 Trustworthiness
Domain 2: Study design			
Theoretical framework			
9.	Methodological orientation and Theory	What methodological orientation was stated to underpin the study? e.g., *grounded theory, discourse analysis, ethnography, phenomenology, qualitative content analysis*. Qualitative content analysis.	3.1 Aim, 3.2 Design, 3.5 Data analysis, 3.7 Trustworthiness
Participant selection			
10.	Sampling	How were participants selected? e.g., *purposive, convenience, consecutive, snowball*. Convenience and purposeful sampling.	3.3 Participants
11.	Method of approach	How were participants approached? e.g., *face‐to‐face, telephone, mail, email*. Telephone and e‐mail.	3.3 Participants
12.	Sample size	How many participants were in the study? 14.	3.3 Participants
13.	Non‐participation	How many people refused to participate or dropped out? Reasons? Nine declined or did not respond to further invitation with the reason of not being interested.	3.3 Participants
Setting			
14.	Setting of data collection	Where was the data collected? e.g., *home, clinic, workplace*. All participants were in their homes during the interviews with video communication.	3.4 Data collection, 5.1 Limitations
15.	Presence of non‐participants	Was anyone else present besides the participants and researchers? The emerging adults living with type 1 diabetes were present in the background when performing two of the interviews.	5.1 Limitations
16.	Description of sample	What are the important characteristics of the sample? e.g., *demographic data, date*. Participants gender, age, diabetes management duration, city of residence, previous experience of type 1 diabetes in the family and experience of working as a healthcare professional, occupation and if they co‐habited the emerging adult living with type 1 diabetes.	3.3 Participants, 3.7 Trustworthiness, 5.1 Limitations
Data collection			
17.	Interview guide	Were questions, prompts, guides provided by the authors? Was it pilot tested? Interview guide with open‐ended and probing questions were used. The interview guide was not pilot tested.	3.4 Data collection, Table 1
18.	Repeat interviews	Were repeat interviews carried out? If yes, how many? No.	
19.	Audio/visual recording	Did the research use audio or visual recording to collect the data? Both audio and visual recording.	3.4 Data collection
20.	Field notes	Were field notes made during and/or after the interview or focus group? No.	
21.	Duration	What was the duration of the interviews or focus group? 21–66 min.	3.4 Data collection
22.	Data saturation	Was data saturation discussed? Yes.	3.4 Data collection, 3.7 Trustworthiness
23.	Transcripts returned	Were transcripts returned to participants for comment and/or correction? No.	
Domain 3: Analysis and findings			
Data analysis			
24.	Number of data coders	How many data coders coded the data? One to three researchers (SO, MB, ÅH) coded data from interviews.	3.5 Data analysis
25.	Description of the coding tree	Did authors provide a description of the coding tree? The findings are presented with an overview in a table.	4 Findings, Table 2
26.	Derivation of themes	Were themes identified in advance or derived from the data? The themes were interpreted and abstracted from the data.	3.5 Data analysis, 4 Findings, 5 Discussion
27.	Software	What software, if applicable, was used to manage the data? No software was used.	3.5 Data analysis
28.	Participant checking	Did participants provide feedback on the findings? No.	
Reporting			
29.	Quotations presented	Were participant quotations presented to illustrate the themes/findings? Was each quotation identified? E.g. *participant number*. Quotations are presented together with a participant number.	4 Findings
30.	Data and findings consistent	Was there consistency between the data presented and the findings? Yes.	4 Findings, 5 Discussion
31.	Clarity of major themes	Were major themes clearly presented in the findings? Yes, a main theme and themes, further subthemes are presented.	4 Findings
32.	Clarity of minor themes	Is there a description of diverse cases or discussion of minor themes? Yes.	4 Findings

*Note:* Reference for the Reporting Method: Tong et al. (2007).
